# Integrative Proteome-Wide Mendelian Randomization and Multi-Omics Analysis Identify ADM and CFH as Candidate Genes for Osteoarthritis

**DOI:** 10.3390/biomedicines14092096

**Published:** 2026-09-17

**Authors:** Haoyang Li, Dongliang Gong, Jun Yang, Zixiang Wang, Junlei Lv, Changan Guo

**Affiliations:** 1 Department of Orthopedics, Zhongshan Hospital, Fudan University, Shanghai 200032, Chinawzx524381459@163.com (Z.W.);; 2 Department of Orthopedics, Qingpu Hospital Affiliated to Fudan University, Shanghai 201700, China

**Keywords:** osteoarthritis, plasma proteome, mendelian randomization, multi-omics, candidate genes

## Abstract

**Background**: Osteoarthritis (OA) is a prevalent degenerative joint disease lacking effective disease-modifying therapies, which necessitates the discovery of key genes for mechanistic exploration and therapeutic development. **Methods**: We integrated three large-scale cis-protein quantitative trait locus datasets and two OA genome-wide association study summary statistics to screen candidate proteins by two-stage proteome-wide Mendelian randomization (MR). Causal association reliability was validated via summary-data-based Mendelian randomization (SMR) and Bayesian colocalization analyses. A phenome-wide association study (PheWAS) was performed to evaluate potential pleiotropic effects of the candidates. Subsequently, transcriptomic and single-cell RNA sequencing datasets were employed to evaluate the candidate genes’ expression stability, classification efficacy in the in vitro models of OA, cell-specific enrichment, and pseudotime expression dynamics in cartilage. Finally, drug repurposing potential was explored by integrating drug–gene interaction database searches and molecular docking. **Results**: Two-stage cis-pQTL MR combined with cis-eQTL-based SMR analysis identified 14 plasma proteins with consistent effects at the protein and transcript levels. RNA-seq revealed that *adrenomedullin (ADM)* and *complement factor H (CFH)* were upregulated in two in vitro models of OA, and both genes exhibited favorable classification efficacy in these models. Bayesian colocalization analysis provided evidence of shared causal variants for *ADM*, and PheWAS did not detect significant pleiotropic associations for *ADM* or *CFH* across the tested phenotypes. Single-cell analysis indicated that *ADM* was enriched in pre-fibrocartilage chondrocytes with biphasic pseudotime expression, whereas *CFH* was widely expressed across chondrocyte subsets. Database screening identified 15 potential drugs for *ADM* and 6 for *CFH*. **Conclusions**: Combining MR, multi-omics and pharmacological evidence, we prioritized *ADM* and *CFH* as OA candidate genes.

## 1. Introduction

Osteoarthritis (OA) is a prevalent degenerative joint disorder characterized by articular cartilage degradation, abnormal subchondral bone remodeling, osteophyte formation, and synovial inflammation [1]. Persistent pain and functional limitations severely impact patients’ quality of life. Current epidemiological data show that more than 500 million individuals are affected worldwide, accounting for 7% of the global population [2]. This prevalence exhibits a persistent upward trajectory driven by population aging. Recognized as a principal contributor to disability among older adults, OA is projected to emerge as the leading cause of population-wide disability by 2030 [3,4]. OA presents a mounting public health challenge that requires urgent therapeutic innovation [2].

Current clinical interventions for OA, including physical therapy, pharmacotherapy, and joint replacement procedures, predominantly relieve symptoms rather than halt or reverse disease progression [2,5,6]. Emerging therapeutic strategies, such as trace element-based interventions [7], stem cell therapies [8], and biologic agents targeting inflammatory cytokines [6], have also been explored; however, they face the same fundamental limitation of failing to modify the underlying disease course. The root cause of this therapeutic bottleneck lies in the insufficient elucidation of the key molecular mechanisms and core genes governing OA pathogenesis. Therefore, identifying core molecular drivers of OA is essential for breaking through the constraints of current symptomatic approaches and advancing the development of disease-modifying osteoarthritis drugs (DMOADs) [9,10,11].

Proteins, as core executors of biological functions, serve not only as key regulatory mediators in disease pathogenesis but also as important potential targets for drug development [12,13]. Therefore, elucidating the causal relationships among genetic variations, protein expression, and disease risk is a crucial prerequisite for identifying stable and reliable OA molecular drivers and advancing targeted drug development [14].

Traditional observational studies are limited by high population heterogeneity, complex pathological processes, and susceptibility to confounding factors, making it difficult to establish definitive causal links between molecular drivers and OA. The rapid advancement of high-throughput sequencing and multi-omics technologies has provided powerful tools for mechanistic studies of complex diseases. Mendelian randomization (MR), which uses genetic variants as instrumental variables, effectively overcomes the biases caused by confounding factors and reverse causation inherent in conventional observational studies, and has thus become a well-established method for inferring causal relationships between exposures and disease outcomes [15,16]. Integration of protein quantitative trait loci (pQTL) data with the MR framework, termed proteome-wide Mendelian randomization (PWMR), enables systematic evaluation of causal associations between circulating plasma protein levels and disease risk, facilitating the identification of potential molecular drivers [12,17,18]. Furthermore, complementary application of methods such as summary data-based Mendelian randomization (SMR), Bayesian colocalization analysis, and phenome-wide association studies (PheWAS) can further validate the robustness of causal associations, explore underlying biological mechanisms, and evaluate potential pleiotropy of candidate molecular drivers.

Although several MR studies have explored the associations between specific proteins and OA, systematic proteome-wide causal inference studies based on large-scale, multi-cohort pQTL data remain limited. Furthermore, comprehensive evaluations of molecular drivers regarding their cell-specific expression in cartilage tissue, druggable assessment, and genetic pleiotropic characteristics are lacking.

This study employed a multi-dimensional integrated analytical strategy to systematically identify high-confidence plasma proteins causally associated with OA. Two-stage MR, SMR, colocalization analysis, and PheWAS were utilized to screen candidates through verifying causal effects and assessing pleiotropy. Subsequently, transcriptomic data and single-cell RNA sequencing (scRNA-seq) data were used to systematically characterize the expression profiles, classification efficacy in the in vitro models of OA, and temporal expression patterns of candidate genes in OA cartilage tissue and specific cell subsets. Finally, drug–gene interaction analysis and molecular docking were performed to explore the repurposing potential of existing drugs for these candidate genes. This multi-dimensional analytical framework, which integrates multiple lines of genetic, transcriptomic, single-cell and pharmacological evidence, offers an innovative strategy for prioritizing core molecular drivers of OA. It not only advances mechanistic insights into OA pathogenesis, but also lays a foundation for developing novel treatment strategies for OA.

## 2. Materials and Methods

### 2.1. Study Design

**Figure 1:**
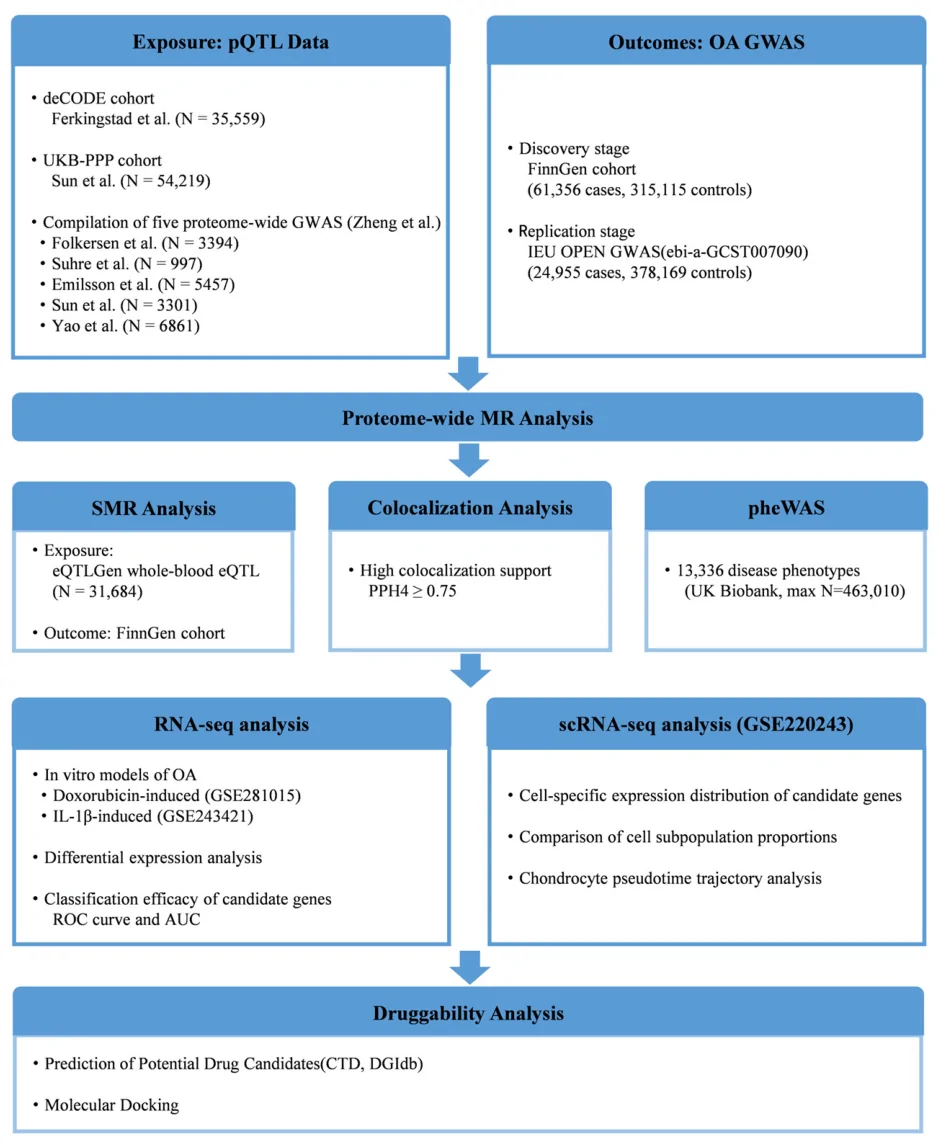
Flowchart of the study design. pQTL, protein quantitative trait loci; OA, osteoarthritis; MR, mendelian randomization; SMR, summary-data-based mendelian randomization; pheWAS, phenome-wide association study; PPH4, Posterior probability of hypothesis 4; IL-1β, interleukin-1β; ROC, receiver operating characteristic; AUC, area under the curve; CTD, Comparative Toxicogenomics Database; DGIdb, Drug–Gene Interaction Database.

First, we integrated pQTL data from three large-scale proteomic studies and performed a two-stage MR validation analysis to identify plasma proteins associated with OA risk, using two independent OA GWAS datasets. Second, SMR analysis and Bayesian colocalization analysis were utilized to verify the reliability of causal associations. PheWAS was used to assess the pleiotropy of candidates. Furthermore, transcriptomic and scRNA-seq data from the Gene Expression Omnibus (GEO) database were utilized to evaluate the expression stability, classification efficacy in the in vitro models of OA, cell-type-specific enrichment, and pseudotime expression dynamics of candidate genes in OA cartilage tissue and specific cell subsets. Finally, we searched drug–gene interaction databases and combined these data with molecular docking to assess the druggability of the candidate genes. Figure 1 shows the flowchart of this study.

### 2.2. Data Sources

We integrated pQTL data from three large-scale proteomic studies, including the DECODE cohort reported by Ferkingstad et al. [13], the UKB-PPP cohort described by Sun et al. [18], and the dataset compiled by Zheng et al. [19] from five previous proteome-wide GWAS studies [20,21,22,23,24]. All participants in these studies were healthy individuals of European ancestry. Additional details about these datasets are available in the original studies. Basic information for the included datasets is provided in Table S1.

Given the proximity of cis-acting expression quantitative trait loci (cis-eQTLs) to their target genes and their more direct functional impact compared to trans-eQTLs, we selected cis-eQTLs as instrumental variables (IVs) for genetic analysis [25,26]. To ensure the selection of high-quality cis-pQTLs, the following stringent criteria were applied: (1) Genome-wide significance with a threshold of *p* < 5 × 10^−8^; (2) exclusion of variants within the major histocompatibility complex (MHC) region (chromosome 6: 26–34 Mb); (3) fulfillment of the independence assumption via linkage disequilibrium (LD) clumping (r^2^ < 0.001); (4) definition of cis-pQTLs as variants located within ±1 Mb of the corresponding protein-coding sequences; (5) exclusion of weak IVs, defined as pQTLs with F-statistics ≤ 10; and (6) exclusion of palindromic single nucleotide polymorphisms (SNPs) and variants with missing data. These selection criteria were implemented to minimize the impact of weak IVs and horizontal pleiotropy on the analysis results, thereby enhancing the validity of causal inference.

Blood cis-eQTL data for SMR validation were obtained from the eQTLGen Consortium database [27]. This database provides summary statistics for cis-eQTLs associated with 16,989 genes, based on 31,684 blood samples from healthy European-ancestry individuals across 37 independent studies. The inclusion criteria were a cis-window of 1 Mb and minor allele frequency (MAF) ≥ 1%.

Two independent OA GWAS summary datasets were employed for the two-stage PWMR analysis. For the discovery stage and SMR analysis, OA GWAS data were obtained from the FinnGen database [28], corresponding to the DF12 data release published on 4 November 2024. This dataset included 21,311,644 genetic variants derived from whole-genome sequencing data of 500,348 Finnish individuals (282,064 females and 218,284 males). For the replication stage, OA GWAS data were obtained from the IEU OpenGWAS database with a GWAS ID of ebi-a-GCST007090 [29]. This dataset comprised 29,999,696 SNPs across 403,124 participants, including 24,955 OA cases and 378,169 healthy controls.

### 2.3. Mendelian Randomization Analysis

We performed two-sample MR analyses to estimate the causal effects between cis-regulated circulating plasma protein levels and OA risk [16,30]. The analytical method was selected based on the number of instrumental variables (IVs). The Wald ratio (WR) method was used when only a single cis-pQTL was available, whereas inverse-variance weighted (IVW) regression was applied for analyses involving two or more independent IVs. The MR results were presented as odds ratios (ORs) with corresponding 95% confidence intervals (95% CIs), standardized per one standard deviation (SD) increase in circulating protein levels. Multiple testing correction was performed using the false discovery rate (FDR) method, with a significance threshold of FDR < 0.05. To ensure the robustness of the results, sensitivity analyses were conducted following IVW analysis. Cochran’s Q test was applied to assess heterogeneity across SNPs, and the MR-Egger intercept was used to evaluate horizontal pleiotropy. A *p*-value > 0.05 was considered indicative of no significant bias. All statistical analyses were performed using the TwoSampleMR package (version 0.6.19) in R software (version 4.4.2). For proteins detected across multiple proteomic cohorts, uniform data harmonization was performed based on UniProt identifiers to standardize cross-cohort protein data. To ensure the rigor and consistency of downstream analytical datasets, only proteins with consistent effect direction, FDR < 0.05, and qualified sensitivity analysis outcomes were included in the candidate set for subsequent validation.

### 2.4. SMR Analysis

To investigate the biological mechanism by which genetic variation influences protein levels through transcriptional regulation and thereby contributes to OA, SMR was employed to evaluate causal associations between plasma protein-coding gene expression and OA risk. The SMR framework tests for pleiotropic links between gene expression and complex traits using summary-level GWAS and QTL data and estimates whether genetic effects on phenotypes are mediated by gene expression [31,32]. The eQTL and OA-related datasets have been described in detail previously. We used the 1000 Genomes Project European (EUR) population as the reference genome. Linkage disequilibrium (LD) was calculated using PLINK v1.9, with a threshold of r^2^ < 0.1. A minor allele frequency (MAF) cutoff of 0.01 was set to exclude low-frequency variants, thereby ensuring the validity of instrumental variables and genetic homogeneity of the study population. The analytical pipeline was performed using SMR v1.3.1 software. Genes meeting a gene-level significance threshold of *p* < 0.05 were retained. The heterogeneity in the dependent instruments (HEIDI) test (P_HEIDI ≥ 0.05) was further applied to distinguish true cis-regulatory effects from pleiotropy or residual LD. The final candidate genes satisfied both the *p*-value significance threshold and the HEIDI homogeneity threshold.

### 2.5. Bayesian Colocalization Analysis

To investigate whether the genetic loci of the genes encoding the proteins corresponding to the identified pQTLs share causal genetic variants with OA, Bayesian colocalization analysis was conducted using the coloc R package (version 5.1.0) [33]. The analysis was restricted to SNPs within a ±1 Mb window around each independent pQTL-associated gene, with summary statistics from OA GWAS used as the outcome dataset. Prior probabilities were set as follows: P1 = 1 × 10^−4^ for SNPs associated only with the pQTL-associated gene, P2 = 1 × 10^−4^ for SNPs associated only with OA, and P12 = 1 × 10^−5^ for SNPs associated with both traits. Colocalization analysis relied on five mutually exclusive hypotheses, with posterior probabilities (PP) calculated for each hypothesis [34]. Specifically, PPH0 represented no association with either trait; PPH1 represented association only with the target gene; PPH2 represented association only with OA; PPH3 represented association with both traits driven by separate causal variants; and PPH4 represented association with both traits mediated by an identical causal variant. PPH4 between 0.5 and 0.75 was regarded as moderate evidence of colocalization, while PPH4 ≥ 0.75 was considered as strong evidence, indicating that genetic variants at the locus likely influence OA susceptibility through regulation of the corresponding protein.

### 2.6. Phenome-Wide Association Studies

To further evaluate the pleiotropy of candidate genes, a PheWAS [35] was conducted across 13,336 disease phenotypes using data from the UK Biobank, with a maximum sample size of 463,010 individuals. The analysis was performed using the AstraZeneca PheWAS Portal [36]. Multiple testing correction was applied using the Bonferroni method, and statistical significance was defined as a corrected *p*-value < 2 × 10^−8^.

### 2.7. RNA-Seq Data Analysis

To further validate the expression stability and classification efficacy of candidate genes in the in vitro models of OA, validation analyses were performed using two transcriptomic datasets from the GEO database, encompassing two in vitro models of OA. The GSE281015 dataset [37] established a chemically induced senescence OA model via doxorubicin treatment of primary mouse articular chondrocytes, while the GSE243421 dataset [38] constructed an inflammaging model by stimulating human knee joint chondrocytes with interleukin-1β (IL-1β). Differential expression analysis was conducted using the limma package in R software (version 4.4.2), with thresholds set at *p* < 0.05 and |log_2_ fold change (log_2_FC)| ≥ 2 to identify differentially expressed genes (DEGs). To evaluate the classification efficacy of candidate genes in the in vitro models of OA, receiver operating characteristic (ROC) curves [39] were plotted, and the area under the curve (AUC) was calculated using the pROC package based on the GSE243421 dataset, which comprised 3 control samples and 3 IL-1β-treated chondrocytes samples. The 95% confidence intervals for AUCs were estimated using non-parametric bootstrap resampling (1000 iterations) to account for the limited sample size. Hub genes with favorable classification efficacy were selected when AUC > 0.85. Subsequently, a nomogram integrating candidate genes was constructed using the rms package to quantitatively assess the predictive performance of the signature in the in vitro OA models.

### 2.8. Single-Cell RNA-Seq Analysis

To elucidate the cell type-specific enrichment and dynamic expression patterns of candidate genes in osteoarthritic cartilage, we utilized the single-cell RNA sequencing (scRNA-seq) dataset GSE220243 [40] from the GEO database, which included 6 OA cartilage biological samples and 6 normal control cartilage biological samples. Analysis was performed using the Seurat package. Quality control criteria were defined as retaining cells with 200–7000 detected genes, excluding low-abundance genes expressed in ≤3 cells, and removing cells with a mitochondrial gene proportion > 25%. After quality control, data normalization was performed using the NormalizeData function (scaling factor = 10,000, normalization.method = “LogNormalize”). We identified 3000 highly variable genes via FindVariableFeatures and scaled expression data with ScaleData. Linear dimensionality reduction was performed via principal component analysis (PCA), followed by batch effect correction with RunHarmony. A shared nearest neighbor graph was constructed using the top 17 principal components, and unsupervised clustering was conducted with FindClusters (resolution = 0.3). Cell dimensionality reduction and visualization were accomplished with the uniform manifold approximation and projection (UMAP) algorithm. Cluster-specific marker genes were identified via FindAllMarkers (parameters: min.pct = 0.25, min.log2FC = 0.50), and cell types were annotated using previously reported canonical marker genes [40]. Subsequently, group-level comparisons of cell subpopulation proportions between OA and control groups were performed based on biological replicate samples. The Wilcoxon rank-sum test was used to compare cell subpopulation proportions between OA and control groups; Bonferroni correction was used for multiple-testing adjustment, with *p* < 0.05 set as the significance threshold. We compared cell subpopulation proportions between OA and control groups to identify significantly differential subpopulations. The cell specific expression distribution of candidate targets across different chondrocyte subpopulations was analyzed, and both Pearson and Spearman correlation coefficients were computed to evaluate pairwise co-expression of candidate genes across single cells. Finally, the Monocle algorithm was used to construct chondrocyte differentiation trajectories and to investigate the dynamic expression changes in candidate genes along pseudotime.

### 2.9. Druggability Analysis

To evaluate the druggability of candidate genes and explore putative drug–gene interactions for the development of OA therapeutics, we systematically searched the Comparative Toxicogenomics Database (CTD) [41] and the Drug–Gene Interaction Database (DGIdb) [42] for drug–gene interaction data related to hub genes. These databases provide comprehensive coverage of approved drugs, clinical trial agents, and experimental compounds. In CTD analysis, an interaction count threshold of ≥5 was set to ensure the reliability of identified drug–gene interactions. Ultimately, drugs with predicted interactions with candidate genes were retrieved from public databases, laying a foundation for drug repurposing.

### 2.10. Molecular Docking

Molecular docking simulations were performed to characterize the in silico binding profiles and potential interaction modes between drugs and candidates, providing structural simulation evidence for subsequent functional verification and potential drug repurposing exploration [43]. Three-dimensional (3D) structural data for drugs were retrieved from the PubChem Compound Database, while structural data for proteins were obtained from the Protein Data Bank (PDB). Docking simulations were conducted using the CB-Dock2 platform [44,45], with binding free energy (kcal/mol) serving as the key parameter to assess the binding stability of ligand–receptor complexes. This tool integrates cavity detection, molecular docking, and homologous template fitting modules to automatically predict potential binding pockets, binding sites, and binding affinity without prior definition of protein active regions. Briefly, the CurPocket curvature-based algorithm was first applied to identify potential druggable cavities on target proteins and calculate the center and size of each pocket. The docking box was customized according to the ligand dimensions and detected cavity characteristics. Subsequently, molecular docking was executed via the embedded AutoDock Vina (version 1.2.0) program to generate optimal protein–ligand docking conformations. Lower binding free energy values indicate greater stability of the formed ligand–receptor complexes. After visual analysis of the docking results, candidate drugs were ranked according to their binding free energy values.

## 3. Results

### 3.1. Causal Plasma Proteins for OA Identified by Proteome-Wide MR

**Figure 2:**
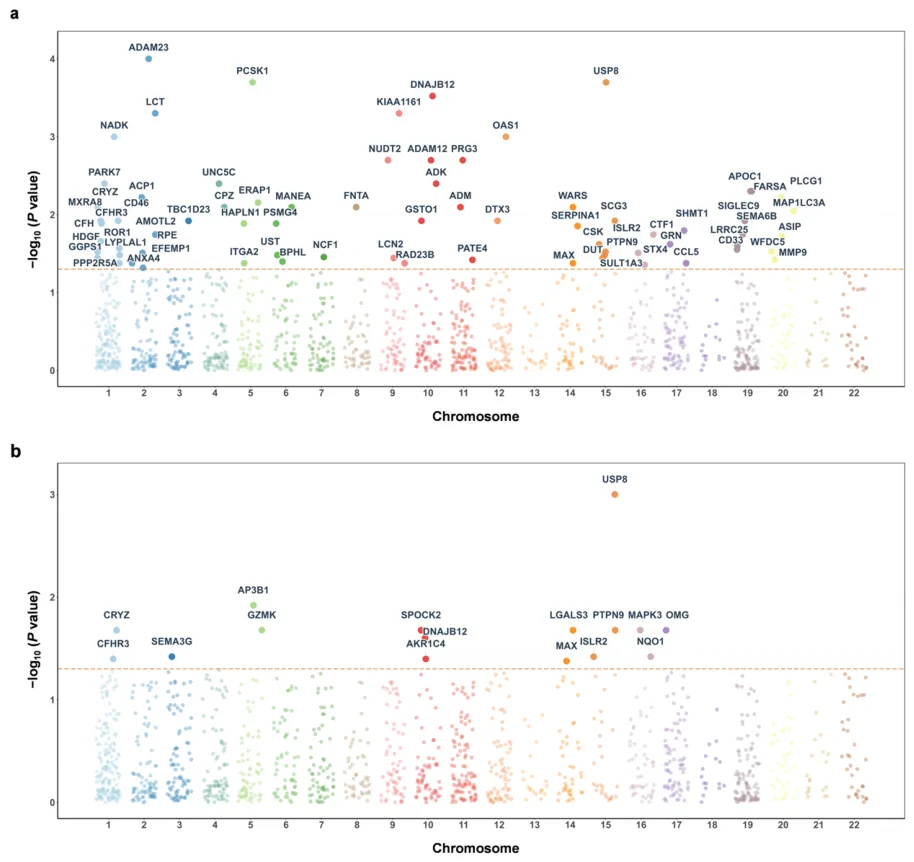
MR analysis results in the discovery stage (**a**) and replication stage (**b**). The horizontal dashed line marks the significance threshold of FDR < 0.05. For the same protein detected in multiple cohorts, only the independent signal with the lowest *p*-value was selected for visualization to avoid redundancy; all eligible analytical results were fully retained in Supplementary Table S5.

To systematically identify candidate molecular targets for OA, genetic instruments were extracted from three large-scale proteome-wide GWAS. The DECODE cohort [13] identified 6214 cis-pQTLs corresponding to 1700 plasma proteins, the UKB-PPP cohort [18] detected 5369 cis-pQTLs for 1883 plasma proteins, and the proteomic GWAS dataset [19] yielded 735 cis-pQTLs for 730 plasma proteins (Tables S2–S4). A two-stage MR approach was employed to evaluate causal relationships between circulating plasma proteins and OA risk, with the discovery stage utilizing summary-level OA GWAS data from the FinnGen cohort and the replication stage adopting independent OA GWAS data published by Tachmazidou et al. In the discovery stage, 71 plasma proteins (Figure 2a and Table S5) showed significant associations with OA susceptibility (FDR-corrected *p* < 0.05), and the subsequent replication stage identified 16 proteins with significant associations (Figure 2b and Table S5); detailed information on the genetic instruments for each protein and on the results of sensitivity analyses are provided in Table S5. Six proteins (PTPN9, DNAJB12, CFHR3, MAX, USP8, ISLR2) consistently met significance thresholds with concordant effect estimates. Elevated levels of PTPN9, CFHR3, MAX, and ISLR2 were inversely associated with OA risk, indicating potential protective roles. Conversely, DNAJB12 and USP8 were associated with an increased OA risk. CRYZ was excluded due to inconsistent effect directions between discovery (OR = 1.026, 95% CI: 1.013–1.040) and validation phases (OR = 0.874, 95% CI: 0.819–0.934). Sensitivity analyses revealed no evidence of horizontal pleiotropy (MR-Egger intercept *p* > 0.05) or heterogeneity (Cochran’s Q *p* > 0.01), supporting the robustness of causal estimates. To comprehensively capture potential OA-relevant functional molecules while retaining biologically meaningful candidates that may exhibit stage-specific signals, we combined all significant proteins from both stages and removed duplicates, yielding 80 unique plasma proteins for subsequent multi-omic SMR and validation analyses. This approach was prioritized over restricting the input to only the six proteins that achieved consistent significant effects and concordant effect directions across both stages.

### 3.2. SMR and Cross-Omic Consistency Validation Identify Genes Associated with OA Risk

**Figure 3:**
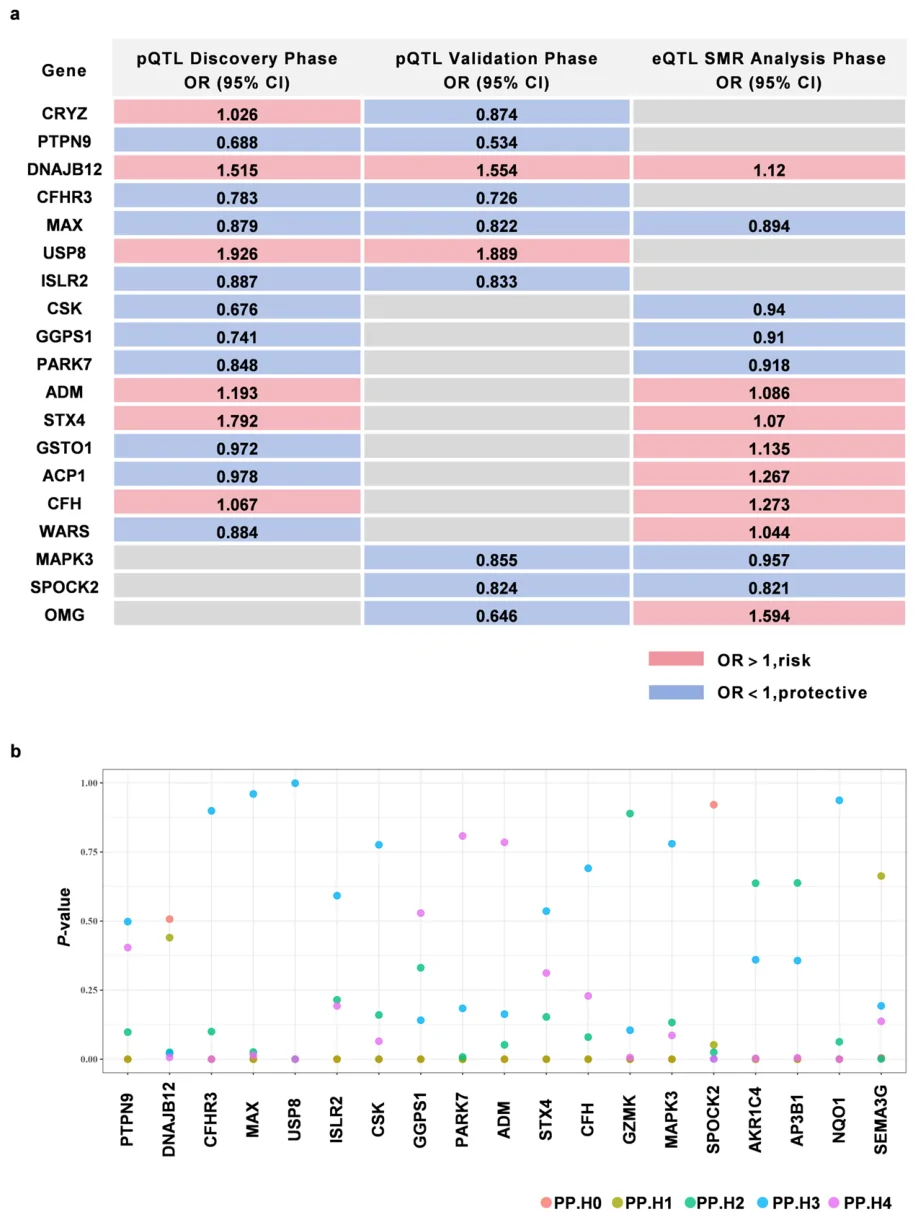
Cross-omic consistency and colocalization evidence of OA risk genes. (**a**) Odds ratios (ORs) of OA-associated candidate genes across three analytical phases: pQTL Discovery Phase, pQTL Validation Phase, and eQTL SMR Analysis Phase. Red shaded cells denote OR > 1, blue shaded cells denote OR < 1, and gray shaded cells represent missing or unavailable data in the corresponding analytical stage. (**b**) Scatter plot illustrating posterior probability (PP) distributions of OA candidate genes under five colocalization hypotheses (PP.H0–PP.H4). The x-axis lists individual OA candidate genes, and the y-axis corresponds to PP values for each colocalization hypothesis.

To investigate the transcriptional regulatory basis underlying the association between plasma proteins and OA, SMR analysis was further performed using cis-eQTLs from the eQTLGen Consortium, combined with the heterogeneity in dependent instruments (HEIDI) test to exclude horizontal pleiotropy. Using a screening threshold of *p* < 0.05 for the SMR test and *p* > 0.05 for the HEIDI test, 384 cis-eQTL-regulated genes associated with OA risk were identified (Table S6). To enhance the robustness of the results, a stringent cross-omics consistency filtering strategy was applied. First, only genes showing significant associations in at least two QTL datasets were retained, leading to the initial identification of 19 candidate genes. Subsequent verification of consistent effect directions led to the exclusion of five genes (*CRYZ*, *GSTO1*, *ACP1*, *WARS* and *OMG*) with discordant effect directions. Ultimately, 14 genes successfully passed combined validation using eQTL and pQTL datasets, with consistent effect directions across different datasets (Table S6 and Figure 3a). These results suggest that these genes may participate in OA pathophysiological processes via multi-omic regulatory pathways spanning transcriptional and protein levels.

### 3.3. Bayesian Colocalization of OA Candidate Genes

To eliminate confounding by genetic pleiotropy induced by LD, Bayesian colocalization analysis was performed on the 14 candidate genes validated through cross-omics validation, aiming to assess the probability that QTLs and the OA phenotype share the same causal variants (Figure S1). The strength of colocalization evidence was classified into three tiers based on the posterior probability PP.H4. Strong colocalization was defined as PP.H4 ≥ 0.75, moderate colocalization as 0.5 ≤ PP.H4 < 0.75, and weak colocalization as PP.H4 < 0.5 [46]. Strong colocalization evidence was observed for *PARK7* (PP.H4 = 0.808) and *ADM* (PP.H4 = 0.785), suggesting that the QTLs of these two genes and OA susceptibility may be driven by the same causal variant (Table S8 and Figure 3b). *GGPS1* (PP.H4 = 0.529) exhibited a moderate colocalization signal (Table S8 and Figure 3b). These findings further support the existence of shared causal variants between the candidate genes and OA susceptibility, strengthening the robustness of the multi-omics analysis results.

### 3.4. PheWAS Analysis of Candidate Genes for OA

**Figure 4:**
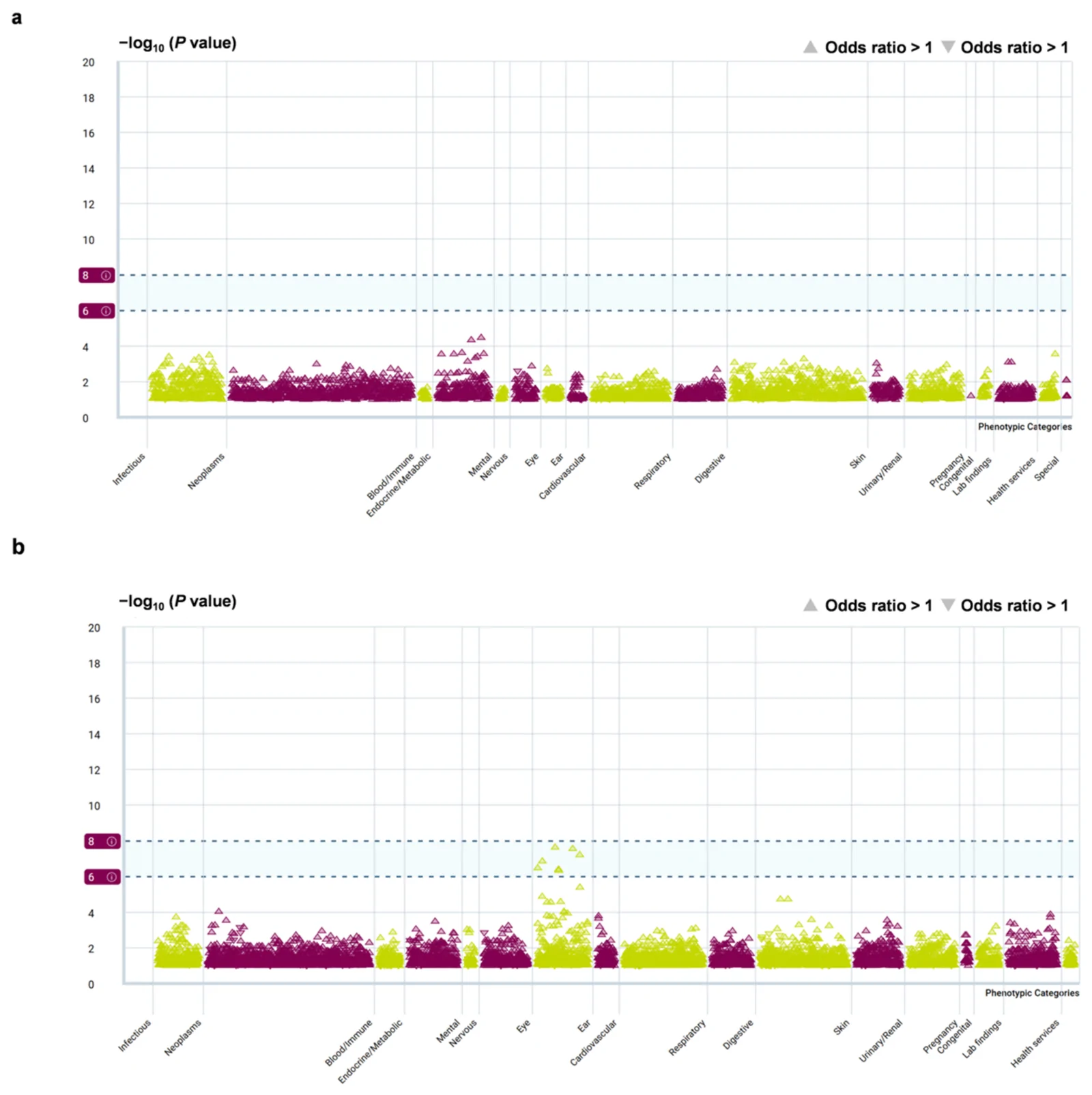
PheWAS of OA candidate genes ADM and CFH. PheWAS results are shown for OA candidate genes ADM (**a**) and CFH (**b**). The y-axis shows −log_10_(*p*-value), and the x-axis represents phenotypic categories from 13,336 UK Biobank disease phenotypes. Upward triangles (△) indicate OR > 1 (risk effect), and downward triangles (▽) indicate OR < 1 (protective effect). The upper blue dashed line marks the significant threshold at −log_10_(*p*-value) = 8, and the lower blue dashed line marks the suggestive threshold at −log_10_(*p*-value) = 6.

To assess the potential pleiotropic risk of the 14 candidate genes, PheWAS was performed using 13,336 disease phenotypes from the UK Biobank database, with a statistical significance threshold set at 1 × 10^−8^. The results showed that none of the 14 candidate genes were significantly associated with any disease phenotype (Figure 4 and Figure S2, Table S9–S22).

### 3.5. External Validation of OA Candidate Genes

**Figure 5:**
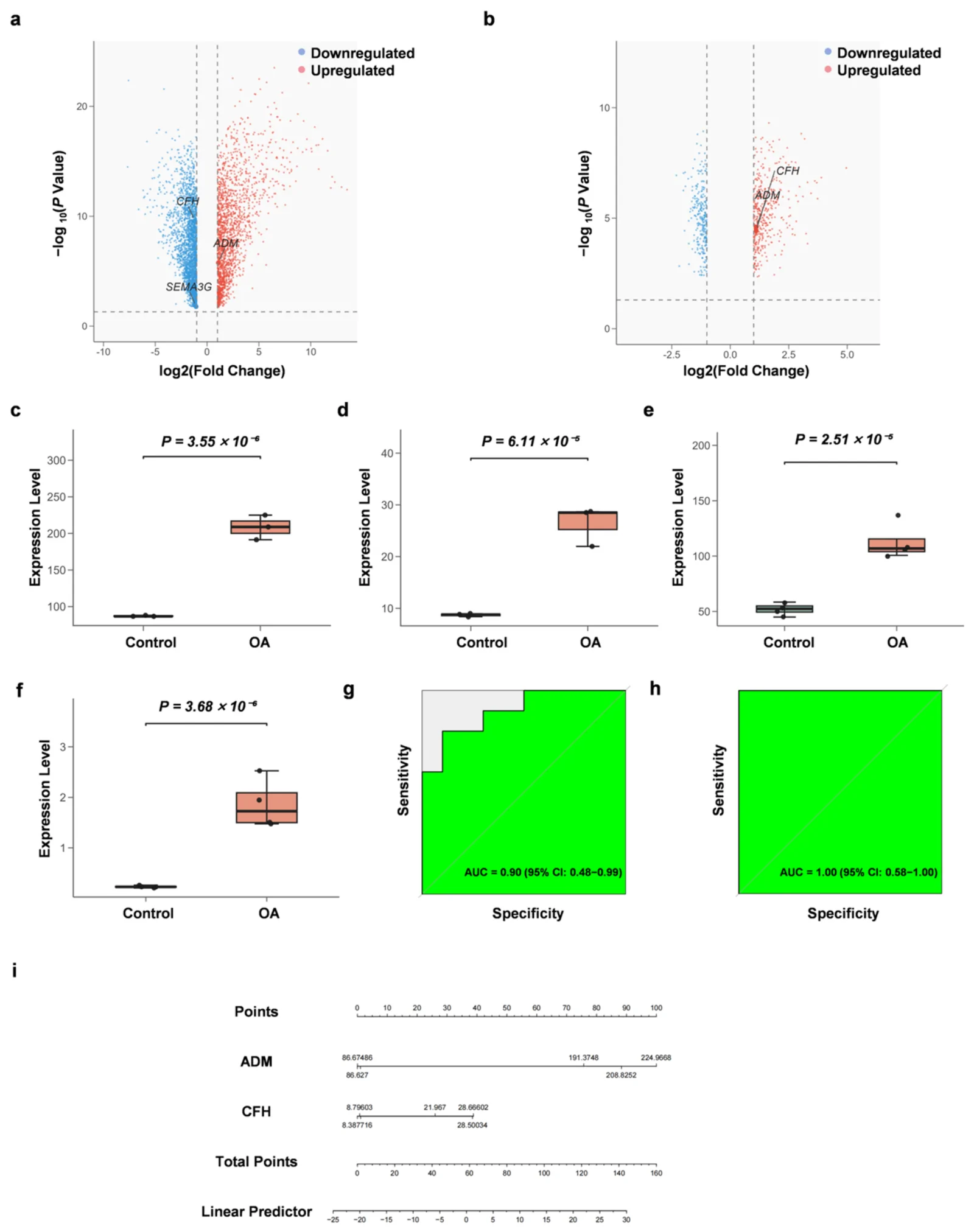
External validation of OA candidate genes. External validation of 14 OA candidate genes using two independent GEO datasets; only *ADM* and *CFH* showed significantly higher expression in the in vitro models of OA than in controls across both datasets. (**a**,**b**) Volcano plots of differential expression (red: upregulated; blue: downregulated). (**c**–**f**) Box plots of *ADM* and *CFH* expression in GSE243421 (**c**,**d**) and GSE281015 (**e**,**f**). (**g**,**h**) ROC curves for *ADM* and *CFH*. (**i**) Nomogram with *ADM* and *CFH* as hub genes for predictive performance in the in vitro models of OA.ROC, receiver operating characteristic; AUC, area under the curve.

To validate the expression stability of candidate genes in the in vitro models of OA, external validation was performed using two independent datasets from the GEO database: an IL-1β-induced inflammatory senescence model of OA (GSE243421) and a doxorubicin-induced chemical senescence model (GSE281015). Among the 14 candidates, only *ADM* and *CFH* displayed concordant expression alterations across the two validation datasets (Figure 5a,b). In GSE243421, *ADM* exhibited log_2_FC = 1.17 (adjusted *p* = 3.55 × 10^−6^) and *CFH* exhibited log_2_FC = 1.76 (adjusted *p* = 6.11 × 10^−5^); in GSE281015, *ADM* exhibited log_2_FC = 1.11 (adjusted *p* = 2.51 × 10^−5^) and *CFH* exhibited log_2_FC = 1.11 (adjusted *p* = 3.68 × 10^−5^). Both genes displayed higher expression levels in OA groups relative to the control groups in both datasets (Figure 5c–f and Tables S23 and S24). These results were highly consistent with the previous predictions, confirming the reliability of their expression differences. A nomogram was generated with *ADM* and *CFH* as hub genes. Each gene was assigned corresponding point values, and the sum of individual scores yielded total points, which could be further converted to linear predictor values for quantitative assessment in in vitro OA models (Figure 5i). ROC curve analysis demonstrated that the AUC exceeded 0.85 for both *ADM* (AUC = 0.90, 95%CI (0.48–0.99), Figure 5g) and *CFH* (AUC = 1.00, 95%CI (0.58–1.00), Figure 5h), indicating discriminatory ability in distinguishing OA-model chondrocytes from normal controls in vitro.

### 3.6. Single-Cell Expression of OA Candidate Genes

**Figure 6:**
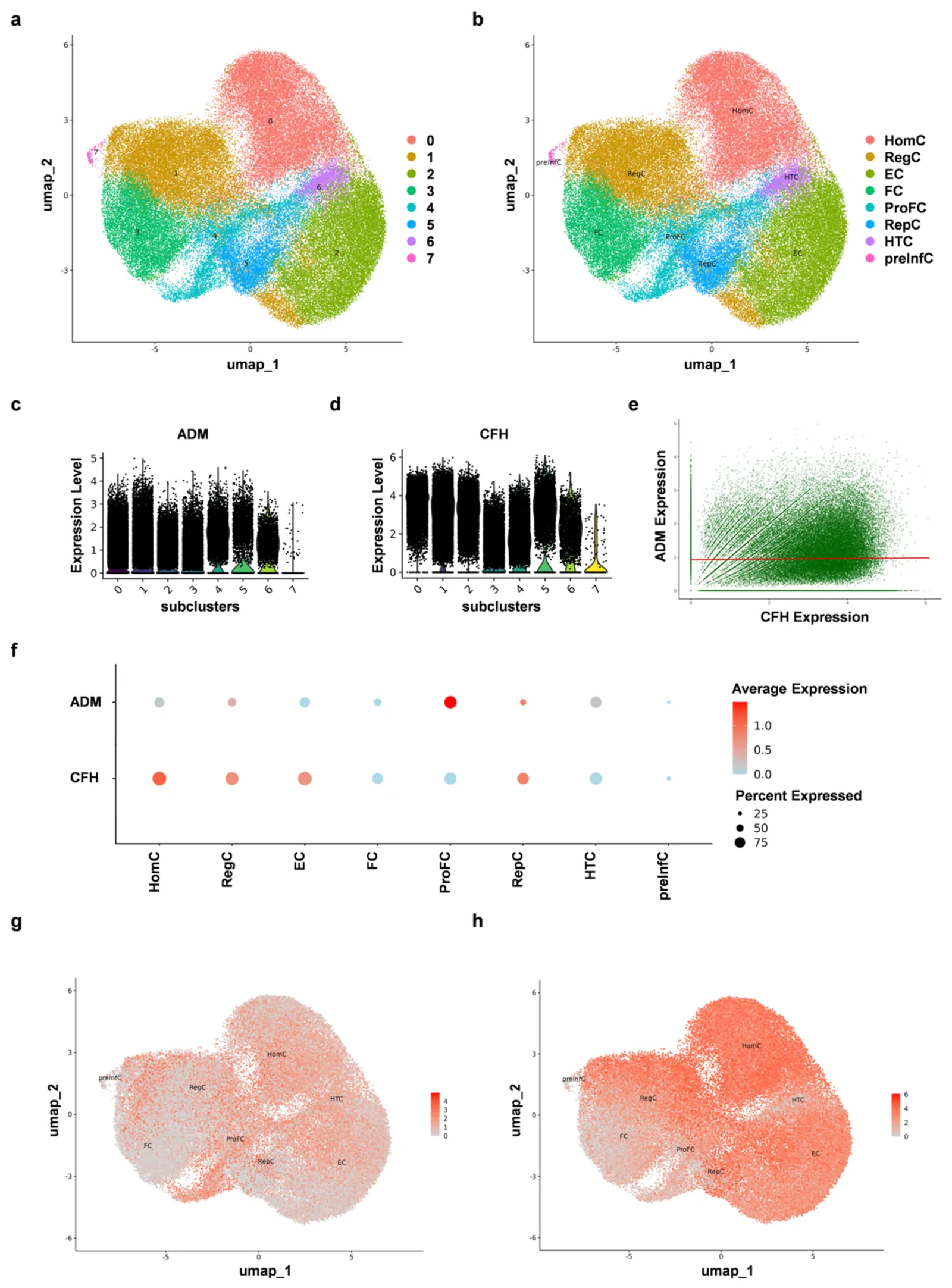
Single-cell landscape of OA chondrocyte subsets. Integrative scRNA-seq analysis of OA candidate genes *ADM* and *CFH* was conducted using the GSE220243 dataset. (**a**) UMAP visualization of the original Seurat clusters. (**b**) Annotation of chondrocyte subtypes, including homeostatic chondrocytes (HomC), regulatory chondrocytes (RegC), effector chondrocytes (EC), fibrocartilage chondrocytes (FC), pre-fibrocartilage chondrocytes (ProFC), reparative chondrocytes (RepC), hypertrophic chondrocytes (HTC), and pre-inflammatory chondrocytes (PrelnfC). (**c**,**d**) Violin plots showing *ADM* and *CFH* expression distributions across cell subtypes. (**e**) Co-expression analysis of *ADM* and *CFH*. (**f**) Bubble plot of cell-type-specific expression: bubble size indicates the percentage of gene-expressing cells, and color gradient represents the average expression level. *ADM* is predominantly expressed in ProFC, while *CFH* is widely distributed across multiple chondrocyte subsets. (**g**,**h**) UMAP projections visualizing the expression levels of *ADM* and *CFH* in individual cells. UMAP: uniform manifold approximation and projection.

**Figure 7:**
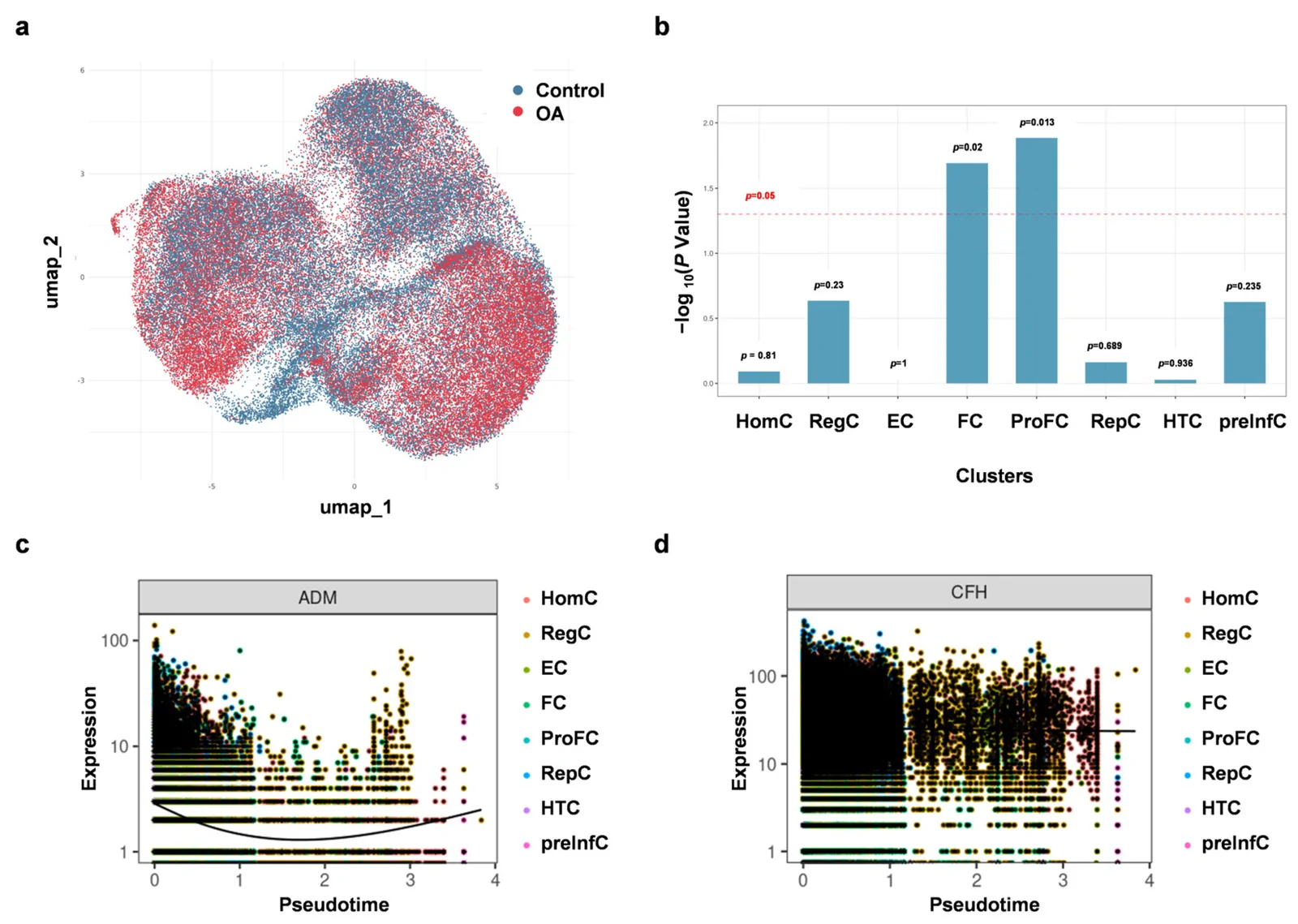
Altered chondrocyte subsets and dynamic *ADM* and *CFH* expression in OA. (**a**) UMAP plot of control (blue) and OA (red) chondrocytes. (**b**) Statistical significance of cluster proportion differences (control vs. OA). Labels show exact *p*-values; dashed line indicates *p* = 0.05. (**c**,**d**) Monocle pseudotime trajectories of *ADM* (**c**) and *CFH* (**d**). *ADM* is ProFC-enriched with biphasic expression; *CFH* is broadly and stably expressed across chondrocyte subtypes.

Integrative analyses were performed using scRNA-seq data from the GSE220243 dataset to characterize the cellular context and underlying pathological regulatory mechanisms of candidate therapeutic targets in OA. Guided by previously reported chondrocyte marker genes [40], unsupervised clustering and cell subtype annotation were performed, leading to the identification of eight distinct chondrocyte clusters: homeostatic chondrocytes (HomC), regulatory chondrocytes (RegC), effector chondrocytes (EC), fibrocartilage chondrocytes (FC), pre-fibrocartilage chondrocytes (ProFC), reparative chondrocytes (RepC), hypertrophic chondrocytes (HTC), and pre-inflammatory chondrocytes (PrelnfC) (Figure 6a,b). Following the Wilcoxon rank-sum test with Bonferroni correction for multiple testing, analysis of cell subpopulation proportions revealed distinct differences between the OA and control groups. Specifically, the proportion of FC was markedly increased (*p* = 0.02), while the proportion of ProFC was significantly decreased (*p* = 0.013) in OA cartilage tissue (Figure 7a,b). Cell-type-specific expression analysis showed that *ADM* was predominantly expressed in the ProFC subset (Figure 6c,f,h), whereas *CFH* was widely distributed across multiple chondrocyte subsets including HomC, RegC, EC and RepC (Figure 6d,f,h), and the expression patterns of other candidate target genes are shown in Figure S3. Additionally, co-expression analysis revealed a significant positive correlation between *ADM* and *CFH* expression levels (Figure 6e). Pseudotime trajectory analysis based on the Monocle algorithm demonstrated that *ADM* expression followed a dynamic pattern of initial downregulation followed by upregulation along chondrocyte differentiation (Figure 7c), while *CFH* maintained stably high expression throughout the entire pseudotime process (Figure 7d). These results clarify the imbalance of chondrocyte subsets in OA, reveal the cell-type-specific expression patterns and dynamic regulatory features of *ADM* and *CFH*, and provide cellular evidence for the therapies against OA.

### 3.7. Druggability Evaluation of the Candidate Genes

To explore putative drug–gene interactions, systematic retrieval was performed using the CTD and DGIdb databases to collect drugs with predicted interactions with the two hub genes (*ADM* and *CFH*). A total of six potential drugs with predicted interactions with *CFH* were identified: Meningococcal Group B Vaccine, eculizumab, ethinyl estradiol, pirinixic acid, cyclosporine, and estradiol (Table 1). For *ADM*, 15 potential drugs were identified, including alteplase, paroxetine hydrochloride, indomethacin, PD-98059, enibarcimab, letrozole, estradiol, phenylephrine, valproic acid, isoproterenol, acetaminophen, tretinoin, cisplatin, cyclic AMP, and ethinyl estradiol (Table 1). Notably, estrogenic compounds (ethinyl estradiol and estradiol) showed predicted interactions with both *ADM* and *CFH*. These findings provide preliminary candidate molecular resources for OA drug repurposing, laying a foundation for future investigation.

### 3.8. Molecular Docking of Candidate Drugs to ADM and CFH

**Figure 8:**
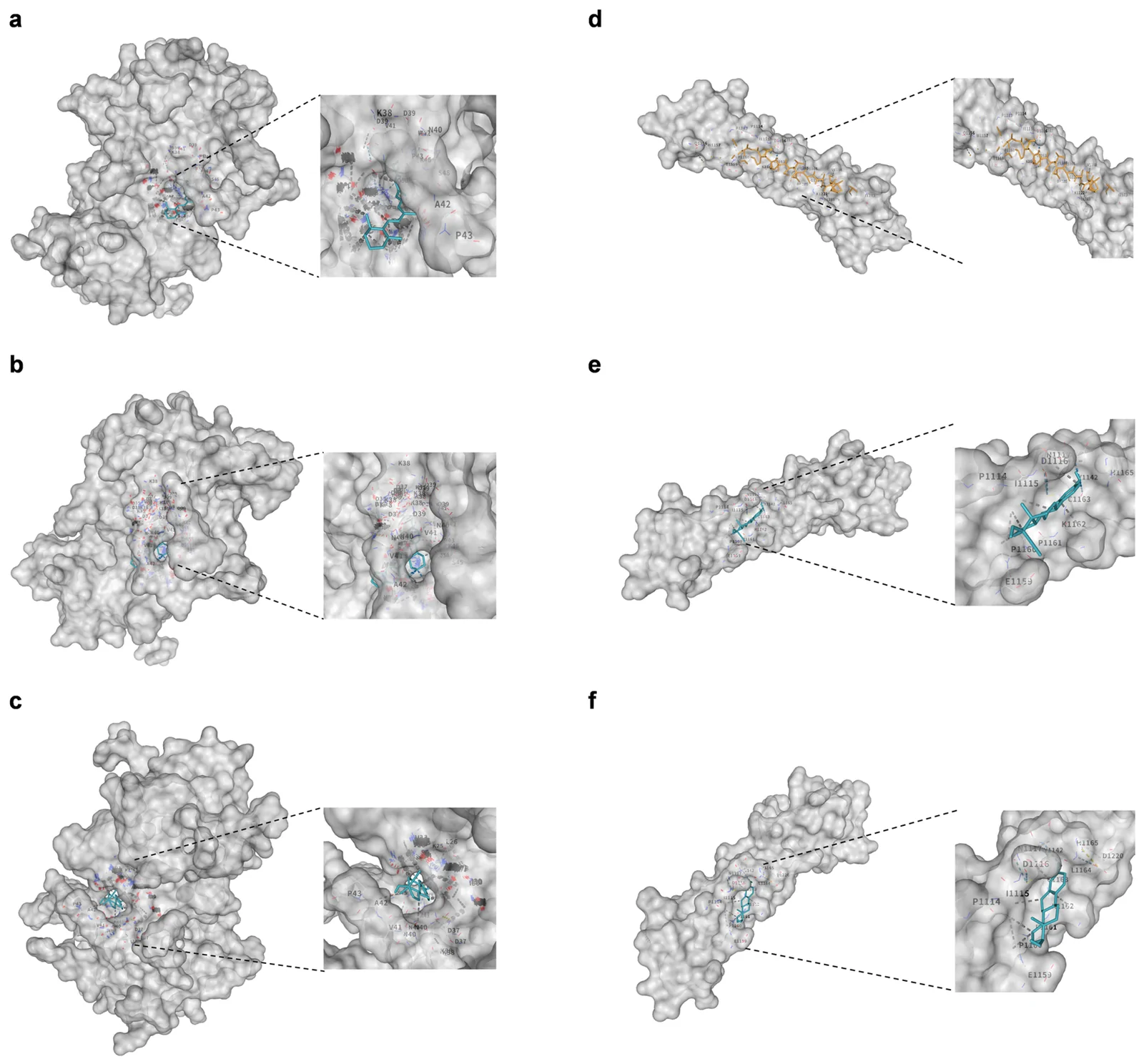
Molecular docking of ADM and CFH with candidate drugs. Molecular docking analysis was performed to evaluate the binding conformations and intermolecular interactions between the two OA hub proteins (ADM and CFH) and candidate drugs. Shown here are the top three highest-affinity docking results for each protein. (**a**) ADM-Tretinoin; (**b**) ADM-Paroxetine hydrochloride; (**c**) ADM-Ethinyl estradiol; (**d**) CFH-Cyclosporine; (**e**) CFH-Ethinyl estradiol; (**f**) CFH-Estradiol.

To further characterize the in silico binding features between candidate drugs and ADM/CFH proteins, molecular docking simulations were performed to comprehensively predict the binding affinity and interaction patterns of ligand–protein complexes [43,47]. For ADM, docking results showed that tretinoin exhibited the strongest binding affinity, with a binding energy of −104.9 kcal/mol. Paroxetine hydrochloride (−103.5 kcal/mol) and ethinyl estradiol (−102.9 kcal/mol) followed, both showing binding energies significantly lower than the typical activity threshold of −5.0 kcal/mol (Figure 8a–c and Table S27). For CFH, cyclosporine showed the optimal computationally predicted binding potential with a binding energy of −9.4 kcal/mol, which was below the −5.0 kcal/mol threshold. Ethinyl estradiol (−6.4 kcal/mol), estradiol (−6.3 kcal/mol), and pirinixic acid (−5.6 kcal/mol) also displayed favorable in silico binding profiles (Figure 8d–f and Table S27).

Ethinyl estradiol exhibited favorable computationally predicted binding profiles with both ADM (−102.9 kcal/mol) and CFH (−6.4 kcal/mol), making it a dual-gene interacting candidate identified via in silico simulation. In addition, several macromolecular candidates, including enibarcimab and eculizumab, were excluded from docking analysis due to the lack of available high-quality 3D structural information. All compounds with complete structural data were successfully subjected to docking simulation. Detailed binding energy data and molecular interaction information are summarized in Figure S4 and Table S27.

In summary, in silico docking simulations predicted that tretinoin, paroxetine hydrochloride, and ethinyl estradiol possess favorable binding potential toward ADM, while cyclosporine, ethinyl estradiol, estradiol, and pirinixic acid show binding profiles for CFH. These computational outputs provide preliminary compound resources for subsequent functional validation and drug repurposing research, and offer hypothetical structural evidence for exploring dual-gene intervention strategies targeting the ADM/CFH axis in OA research.

## 4. Discussion

OA is a complex degenerative joint disease characterized by polygenic regulation and interconnected molecular pathways. Current clinical medications primarily focus on anti-inflammatory and analgesic effects, with no available agents that can block or even reverse the progression of OA [2,5,6]. Identifying reliable therapeutic targets and effective intervention strategies therefore remains a major clinical challenge.

Leveraging large-scale proteomic GWAS data, this study employed a two-stage MR approach to identify circulating proteins significantly associated with OA risk across two independent OA GWAS cohorts. Through multi-omics integration with cis-eQTL data from the eQTLGen consortium, we identified 14 candidate genes demonstrating concordant effects at both transcriptional and protein levels. Bayesian colocalization analysis further pinpointed shared causal variants between these candidate genes and OA phenotypes, while no significant cross-phenotype associations were observed in the PheWAS screening. Validation using GEO datasets demonstrated that *ADM* and *CFH* were upregulated in two OA chondrocyte senescence models, consistent with the MR findings. The gene-based signature constructed for in vitro OA models showed good classification efficacy, achieving an AUC above 0.85 upon validation using in vitro model datasets. ScRNA-seq analysis revealed that *ADM* was primarily enriched in the ProFC subpopulation, whereas *CFH* displayed a broader expression pattern, and both exhibited dynamic temporal regulatory characteristics. Moreover, co-expression analysis further demonstrated a significant positive correlation between *ADM* and *CFH* expression levels. Computational drug screening via CTD and DGIdb databases identified 15 and 6 compounds with predicted interactions with *ADM* and *CFH*, respectively, with ethinyl estradiol showing favorable computationally predicted binding profiles with both *ADM* and *CFH*. Our multi-layered validation framework integrating multi-omics profiling, cellular localization, and structural pharmacology systematically identified OA-associated genes and potential drugs, providing genetic, cellular, and pharmacological evidence for OA therapeutics.

Using an integrative multi-omics approach, *ADM* and *CFH* were identified as potential susceptibility genes for OA. Their potential as OA biomarkers was preliminarily validated. Integrated analysis of pQTLs and eQTLs further supported that both *ADM* and *CFH* were risk genes for OA. Notably, Bayesian colocalization analysis detected robust colocalization signals and shared causal genetic variants between ADM and OA, supporting a genetic causal link between *ADM* and OA. By contrast, *CFH* did not meet the colocalization criteria, suggesting that it lacks comparable genetic causal evidence for OA pathogenesis. RNA sequencing revealed significantly upregulated expression of *ADM* and *CFH* in two distinct chondrocyte senescence models of OA. ScRNA-seq further showed that *ADM* was predominantly enriched in the ProFC subset. Pseudotime trajectory analysis suggested a dynamic expression pattern of *ADM* during chondrocyte differentiation, with an initial decrease followed by an increase. In contrast, *CFH* was broadly expressed across multiple chondrocyte subsets and maintained consistently high expression levels throughout differentiation. Furthermore, the expression levels of *ADM* and *CFH* were found to be significantly positively correlated. These findings indicate that *ADM* and *CFH* may play distinct regulatory roles in maintaining chondrocyte homeostasis and in the progression of OA pathology.

*ADM*, a member of the amylin/calcitonin gene-related peptide superfamily, is a vasoactive peptide with diverse biological activities. It regulates physiological processes including cell proliferation, differentiation, vasodilation, and hormone secretion, and contributes to cardiovascular diseases such as malignant hypertension and heart failure, as well as various tumors [48,49]. In OA, previous studies have reported elevated *ADM* concentrations in the serum and synovial fluid of patients, levels that positively correlate with Kellgren–Lawrence grade. This correlation implies that ADM may serve as a fluid biomarker for assessing OA severity [50], yet its expression and functional roles within osteoarthritic cartilage remain unreported to date. *CFH* is a major negative regulator of the alternative complement pathway, primarily functioning to accelerate C3 convertase decay and mediate C3b inactivation for maintaining complement system homeostasis [51,52]. Additionally, *CFH* exerts non-canonical functions by regulating endothelial integrity, mitochondrial metabolism, and cell proliferation, thereby influencing tumor cell biological behaviors and retinal cell oxidative stress and energy metabolism [53]. However, the specific role of *CFH* in osteoarthritis remains poorly understood.

Notably, *CFH* has been identified as a specific binding protein of *ADM*, also known as *adrenomedullin binding protein-1 (AMBP-1)* [54,55]. These two proteins directly interact through hydrophobic forces to form a stable complex, exerting bidirectional synergistic regulatory effects. On one hand, *ADM* enhances the complement cofactor activity of *CFH* in a dose-dependent manner by inducing conformational changes, thereby accelerating C3b cleavage to strengthen the blockade of the alternative complement pathway. On the other hand, *CFH* protects *ADM* from enzymatic degradation by *matrix metalloproteinase 2 (MMP2)*, leading to a prolonged *ADM* half-life and increased cyclic AMP (cAMP) production [54,55,56,57,58]. Given that aberrant activation of the alternative complement pathway has been implicated as a driver of cartilage matrix degradation and synovial inflammation in OA [59,60], combined with the expression profiles and cellular distribution patterns of *ADM* and *CFH* characterized in our study, we hypothesize that *ADM* and *CFH* cooperatively modulate OA progression. Beyond their regulatory roles in the alternative complement pathway, *ADM* and *CFH* may also exert non-canonical pro-degenerative effects to facilitate OA pathogenesis.

Collectively, this study establishes a strong association between *ADM* and *CFH* with OA through multi-omics integrated analysis. It reveals their abnormal expression patterns, subpopulation distribution, and dynamic expression characteristics in OA chondrocytes. Based on their specific binding and functional synergy, we propose a mechanistic hypothesis in which the *ADM-CFH* complex participates in OA pathogenesis through a two-stage regulatory mode. This work enhances the understanding of genetic susceptibility and molecular pathological networks in OA. It also provides a crucial theoretical basis and experimental foundation for developing early diagnostic biomarkers and stage-specific targeted intervention strategies.

Based on drug predictions from the CTD and DGIdb databases combined with molecular docking analysis, this study identified multiple candidate drugs for ADM and CFH. Among them, ethinyl estradiol, a compound with predicted interactions with both ADM and CFH, showed particular interest in further investigation. This compound yielded docking binding energies of −102.9 kcal/mol against ADM and −6.4 kcal/mol against CFH, indicating favorable in silico binding profiles. Furthermore, favorable in silico binding profiles were observed for tretinoin against ADM (−104.9 kcal/mol) and cyclosporine against CFH (−9.4 kcal/mol), providing preliminary structural simulation evidence for the potential repurposing of these drugs in subsequent OA research. Although these computational predictions require rigorous in vitro and in vivo experimental validation, our multi-omics analytical framework provides a platform for the rapid preliminary screening of candidate OA-related compounds.

From systematic retrieval based on the published literature, we further inspected potential links between predicted drugs and OA. Evidence for estrogenic compounds (ethinyl estradiol and estradiol) in OA remains inconsistent. Pre-clinical and clinical observations suggest potential favorable effects together with acceptable safety profiles, particularly for post-menopausal or osteoporosis-associated OA, yet supporting data are not sufficiently robust to establish definite therapeutic utility [61]. Loss of estradiol may accelerate chondrocyte senescence, extracellular matrix breakdown and knee cartilage degeneration, whereas estradiol supplementation could mitigate such pathological changes, which may partly inform the sex-biased susceptibility of knee OA among post-menopausal females [62,63]. Among other candidates, indomethacin may modulate chondrocyte inflammation, matrix turnover, apoptosis and autophagy via the PI3K/AKT/mTOR cascade to slow OA-related damage [64], and valproic acid could alleviate LPS-triggered chondrocyte injury through the miR-302d-3p/ITGB4 axis and PI3K-AKT signaling inhibition [65]. By contrast, cyclosporine showed no benefit against lameness in canine OA models, arguing against its potential application in this setting [66]. Cisplatin may contribute to cartilage injury by triggering chondrocyte oxidative stress, apoptosis and pro-inflammatory responses via the LOX-1/p38/NF-κB pathway [67]. No relevant OA-associated records were identified for the remaining predicted compounds. Collectively, these published reports exhibit substantial heterogeneity; our in silico drug–gene interaction predictions require further experimental validation.

The strengths of this study include validating the consistency of candidate genes at the genetic, transcriptional and cellular levels using large-scale GWAS-based MR analysis and multi-omics validation, thereby ensuring the reliability of causal inference. The single-cell atlas reveals the cell-specific expression and dynamic differentiation trajectories of *ADM* and *CFH*, capturing temporal regulatory patterns that cannot be resolved by traditional bulk transcriptomics, thus enabling precise cellular targeting for OA. Integration of drug–gene interaction databases and molecular docking enables in silico identification of candidate drugs for potential drug repurposing. Consistent results across multi-omics multiple populations and multiple cohorts strengthen the evidence for *ADM* and *CFH* as key regulators of OA.

Several limitations should be noted. First, the study population was predominantly of European ancestry, which limits the cross-ethnic generalizability of the findings and future studies should include multi-ethnic cohorts to enhance the generalizability of the results. Second, the analysis focused solely on cis-pQTLs without including trans-pQTLs. Although this approach reduced the risk of violating MR assumptions, it also overlooked more complex regulatory mechanisms underlying protein expression. Third, while PheWAS detected no significant pleiotropic signals, the limited phenotype coverage in the UK Biobank may have missed rare adverse phenotype associations. Fourth, this scRNA-seq analysis relied on standard log-normalized expression without dedicated zero-inflated modeling, which may introduce potential bias for low-expressed genes. Fifth, the ROC analyses were performed in a small cohort, which yielded wide 95% confidence intervals for AUC estimates and thus these findings should be interpreted as exploratory, requiring validation in larger independent cohorts. Additionally, although multi-omics evidence supports the genetic associations of *ADM* and *CFH* with OA and our proposed two-stage mechanism hypothesis can adequately explain the current multi-dimensional observations, several questions remain to be addressed. Future studies should conduct systematic validation and in-depth analysis using in vitro cell function assays, in vivo animal model interventions, protein–protein interaction experiments, and spatial localization techniques.

The findings of this study provide candidate genes and in silico-derived drug repurposing clues for OA research. As hub genes prioritized via multi-omics analytical approaches, *ADM* and *CFH* show predicted druggability. In particular, ethinyl estradiol emerges as a computationally predicted dual-gene candidate, which warrants further experimental exploration.

## 5. Conclusions

In summary, this study employed a multi-omics integrative strategy to identify and validate *ADM* and *CFH* as key genes for OA. Integrating prior functional evidence of *ADM* and *CFH* with the multi-dimensional computational data from this study, we hypothesize that the *CFH-ADM* axis exerts a regulatory effect on the pathogenesis of OA. Through Drug–Gene Interaction Database screening and molecular docking simulations, we identified candidate drugs with favorable in silico binding profiles, among which ethinyl estradiol was notably predicted to interact with both *ADM* and *CFH*. These findings yield novel candidate genes and therapeutic agents for OA research, laying a solid foundation for the development of chondrocyte-targeted therapeutic strategies for OA.

## Figures and Tables

**Table 1:** Druggability evaluation of *ADM* and *CFH*. A systematic retrieval of potential drugs with predicted interactions with *ADM* and *CFH* was performed using the CTD and DGIdb databases.

Targets	Drug	Source	Approved Indications
CFH	Meningococcal Group B Vaccine	DGldb	Approved
	Eculizumab	DGldb	Approved
	Ethinyl estradiol	CTD	Approved
	Pirinixic acid	CTD	Approved
	Cyclosporine	CTD	Approved
	Estradiol	CTD	Approved
ADM	Alteplase	DGldb	Approved
	Paroxetine hydrochloride	DGldb	Approved
	Indomethacin	DGldb	Approved
	PD-98059	DGldb	Not Approved
	Enibarcimab	DGldb	Not Approved
	Letrozole	CTD	Approved
	Estradiol	CTD	Approved
	Phenylephrine	CTD	Approved
	Valproic acid	CTD	Approved
	Isoproterenol	CTD	Approved
	Acetaminophen	CTD	Approved
	Tretinoin	CTD	Approved
	Cisplatin	CTD	Approved
	Cyclic AMP	CTD	Approved
	Ethinyl estradiol	CTD	Approved

## Data Availability

All data supporting the findings are from open-access sources and are included in the Article and its Supplementary Information.

## Supplementary Materials

The following supporting information can be downloaded at https://www.mdpi.com/article/10.3390/biomedicines14092096/s1, Table S1. Details of all data sources in this study; Table S2. Genetic variants of pQTLs from Ferkingstad et al. used in this study; Table S3. Genetic variants of pQTLs from Sun 1 et al. used in this study; Table S4. Genetic variants of pQTLs from Zheng et al. used in this study; Table S5. Mendelian randomization results of cis-pQTLs for osteoarthritis; Table S6. SMR results of cis-eQTLs for osteoarthritis; Table S7. Multi-omic validation of OA risk genes; Table S8. Colocalization analysis results between OA and candidate genes; Table S9. PheWAS results for PTPN9; Table S10. PheWAS results for DNAJB12; Table S11. PheWAS results for CFHR3; Table S12. PheWAS results for MAX; Table S13. PheWAS results for USP8; Table S14. PheWAS results for ISLR2; Table S15. PheWAS results for CSK; Table S16. PheWAS results for GGPS1; Table S17. PheWAS results for PARK7; Table S18. PheWAS results for ADM; Table S19. PheWAS results for STX4; Table S20. PheWAS results for CFH; Table S21. PheWAS results for MAPK3; Table S22. PheWAS results for SPOCK2; Table S23. Results of differential expression analysis of the GSE243421 dataset; Table S24. Results of differential expression analysis of the GSE281015 dataset; Table S25. Results of single-gene GSEA for CFH; Table S26. Results of single-gene GSEA for ADM; Table S27. Molecular docking results of ADM and CFH with candidate drugs; Figure S1. Regional association plots from colocalization analysis of candidate genes and OA; Figure S2. PheWAS of OA candidate genes; Figure S3. Single-cell expression profiles of OA candidate genes across cell subsets; Figure S4. Molecular docking of ADM and CFH with candidate drugs.

## Abbreviations

The following abbreviations are used in this manuscript:

ADM	adrenomedullin
AUC	area under the curve
CFH	complement factor H
CI	confidence interval
cis-eQTL	cis-acting expression quantitative trait locus
cis-pQTL	cis-protein quantitative trait locus
CTD	Comparative Toxicogenomics Database
DGIdb	Drug–Gene Interaction Database
DMOAD	disease-modifying osteoarthritis drug
eQTL	expression quantitative trait locus
FDR	false discovery rate
GEO	Gene Expression Omnibus
GWAS	genome-wide association study
IV	instrumental variable
IVW	inverse-variance weighted
LD	linkage disequilibrium
MAF	minor allele frequency
MHC	major histocompatibility complex
MR	Mendelian randomization
OA	osteoarthritis
OR	odds ratio
PDB	Protein Data Bank
PheWAS	phenome-wide association study
PP	posterior probability
pQTL	protein quantitative trait locus
PWMR	proteome-wide Mendelian randomization
ROC	receiver operating characteristic
scRNA-seq	single-cell RNA sequencing
SMR	summary data-based Mendelian randomization
SNP	single nucleotide polymorphism
UMAP	uniform manifold approximation and projection
WR	Wald ratio
