# Supplementary material for: Integrative Proteome-Wide Mendelian Randomization and Multi-Omics Analysis Identify ADM and CFH as Candidate Genes for Osteoarthritis
Source: Biomedicines. 2026 Sep 17;14(9):2096. doi: 10.3390/biomedicines14092096 (PMC13604318; doi:10.3390/biomedicines14092096)

Figure S1.Regional association plots from colocalization analysis of candidate genes and OA. Panels A-N represent the following genes: PTPN9 (A), DNAJB12 (B), CFHR3 (C), MAX (D), USP8 (E), ISLR2 (F), CSK (G), GGPS1 (H), PARK7 (I), ADM (J), STX4 (K), CFH (L), MAPK3 (M), and SPOCK2 (N).The left bivariate scatter plot shows the correlation between QTL significance (represented as $-\log_{10}(P)$) and OA GWAS significance.The right panel presents regional association plots for the corresponding chromosomal region: the upper subplot displays QTL association signals, and the lower subplot displays OA GWAS association signals. Variants are colored according to their linkage disequilibrium (LD) with the lead variant, with the color bar indicating r^2values.

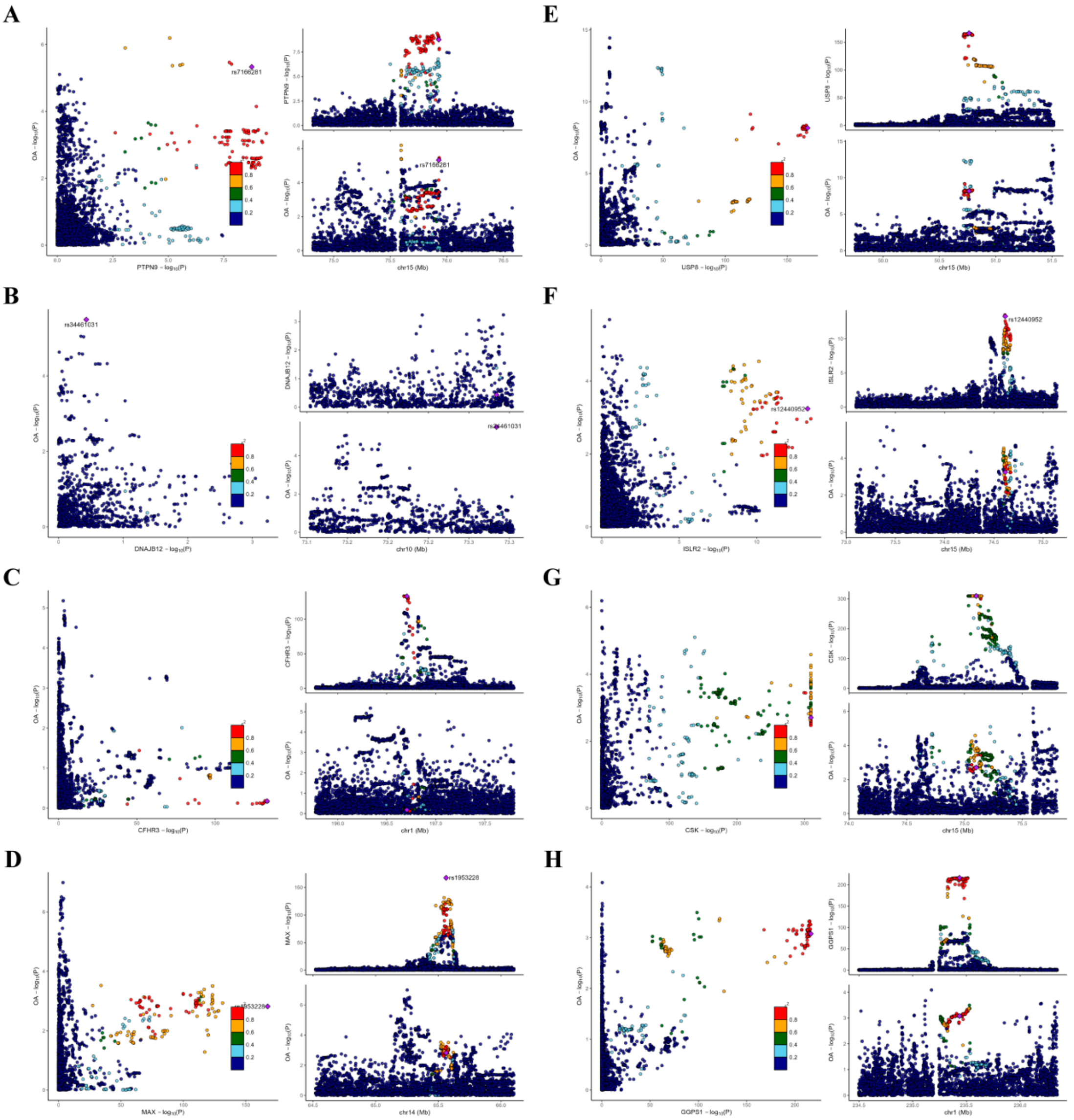
A
B
C
D
E
F
G
H
rs7166281
PTPN9 - log10(P)
chr15 (Mb)
rs34461031
DNAJB12 - log10(P)
chr10 (Mb)
CFHR3 - log10(P)
chr1 (Mb)
rs1953228
MAX - log10(P)
chr14 (Mb)
USP8 - log10(P)
rs12440952
ISLR2 - log10(P)
CSK - log10(P)
GGPS1 - log10(P)
OA - log10(P)

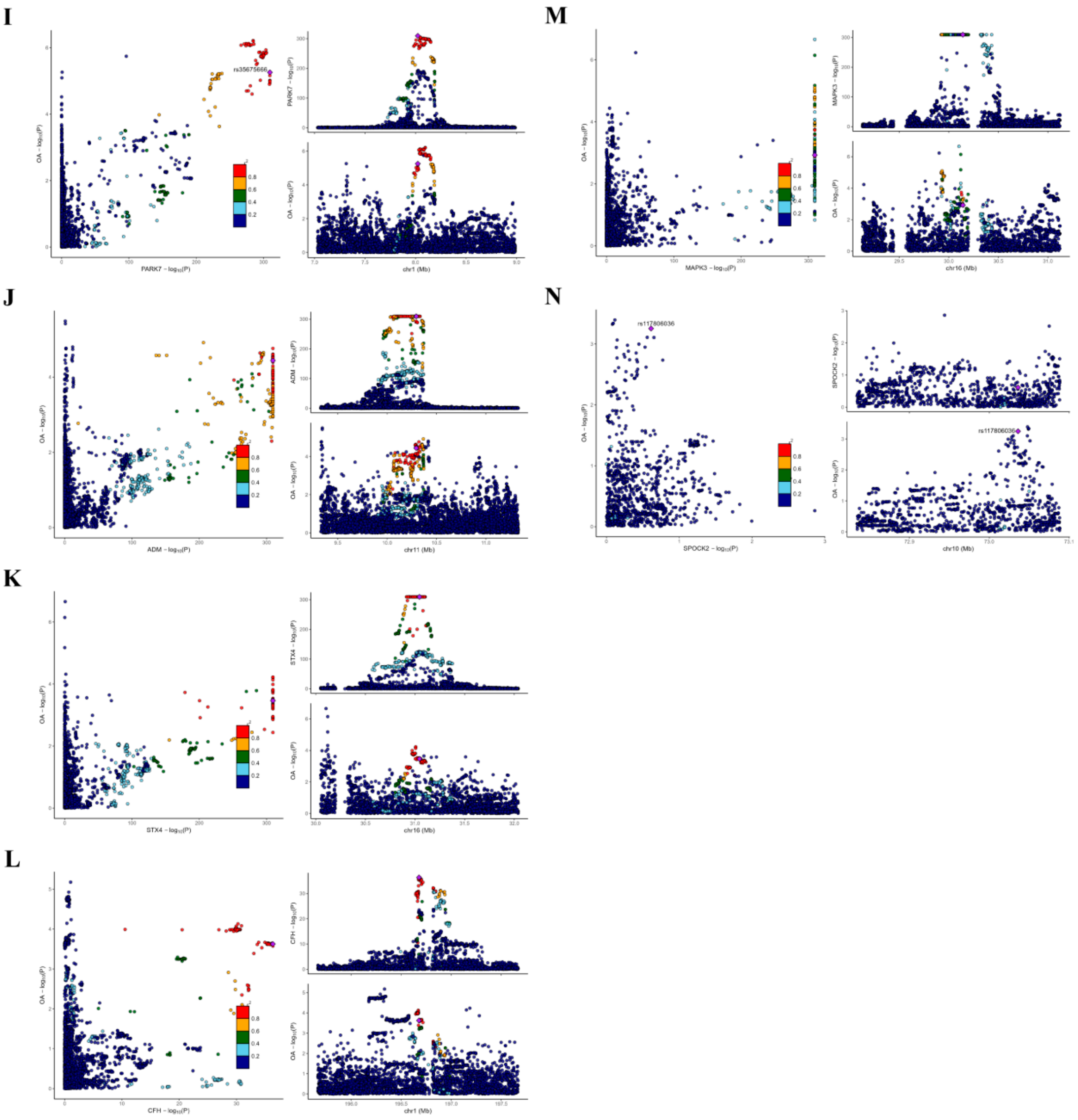

Figure S2. PheWAS of OA Candidate Genes. A-L present pheWAS results for PTPN9 (A), DNAJB12 (B), CFHR3 (C), MAX (D), USP8 (E), ISLR2 (F), CSK (G), GGPS1 (H), PARK7 (I), STX4 (J), MAPK3 (K), and SPOCK2 (L). The y-axis shows $-\log_{10}$(P-value), and the x-axis represents phenotypic categories from 13336 UK Biobank disease phenotypes. Upward triangles (△) indicate OR > 1 (risk effect), and downward triangles (▽) indicate OR < 1 (protective effect). The horizontal dashed line marks the significance threshold of 2×10^{-8}.

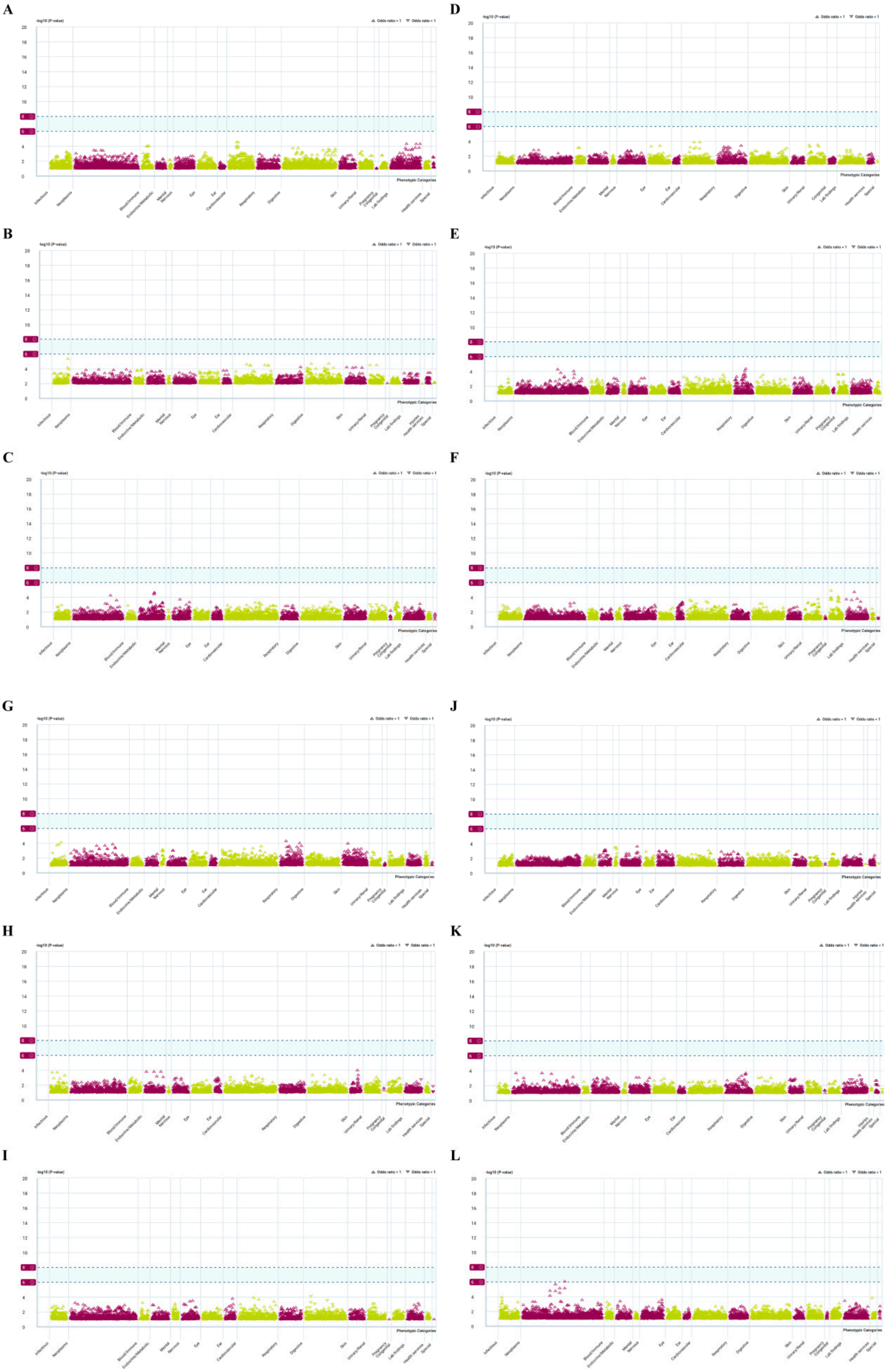
A
B
C
D
E
F
G
H
I
J
K
L
-log10 (P-value)
Odds ratio > 1
Odds ratio < 1
Phenotypic Categories
Infectious
Neoplasms
Blood/Immune
Endocrine/Metabolic
Mental
Nervous
Eye
Ear
Cardiovascular
Respiratory
Digestive
Skin
Urinary/Renal
Pregnancy
Congenital
Lab findings
Health services
Special

Figure S3.Single-cell expression profiles of OA candidate genes across cell subsets.A–G show single-cell expression distributions for STX4 (A), MAX (B), MAPK3 (C), GGPS1 (D), PARK7 (E), AP3B1 (F), and USP8 (G). The y-axis represents normalized gene expression levels, and the x-axis indicates distinct cell subsets. Individual black points denote expression values in single cells, while colored boxplots display the median and distribution range within each subset.

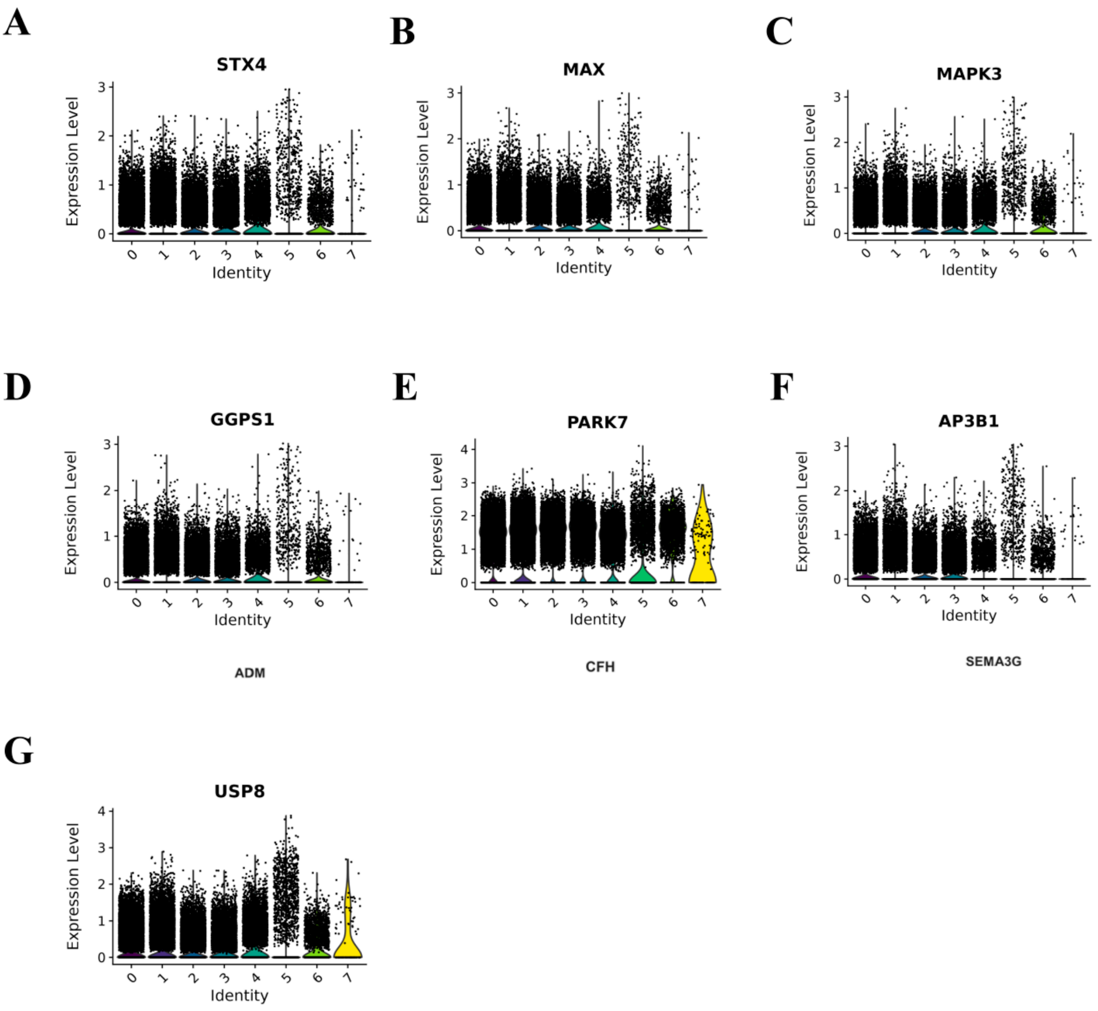

Figure S4. Molecular docking of ADM and CFH with candidate drugs. Molecular docking analysis was performed to evaluate the binding conformations and

intermolecular interactions between the two OA hub genes (ADM and CFH) and candidate drugs. Shown here are the docking results of target genes with corresponding candidate drugs.(A) CFH–pirinixic acid; (B) ADM–Letrozole; (C) ADM–Estradiol; (D) ADM–Phenylephrine; (E) ADM–Valproic Acid; (F) ADM–Isoproterenol; (G) ADM–Acetaminophen; (H) ADM–Cyclic AMP; (I) ADM–Indomethacin; (J) ADM–PD-98059.

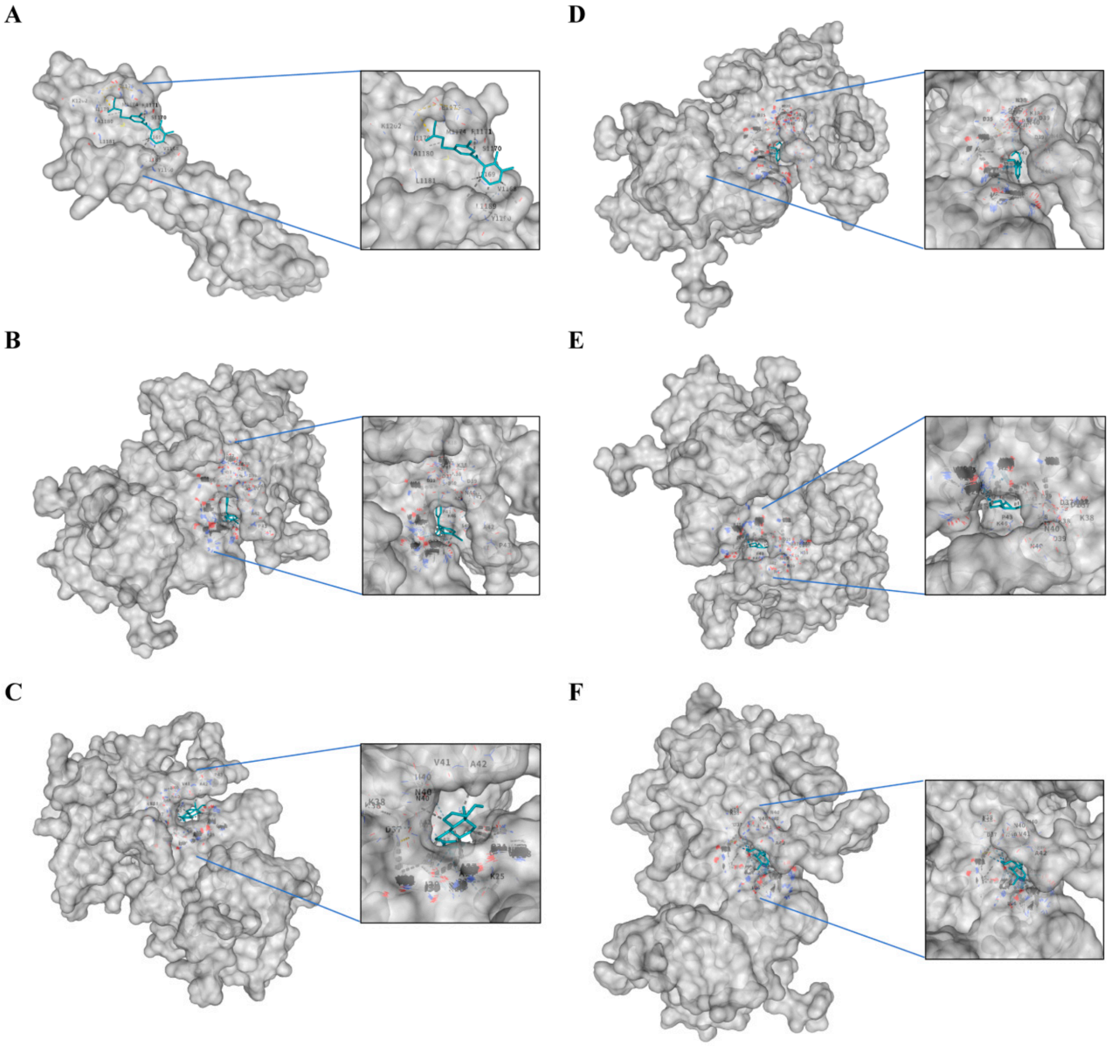

G

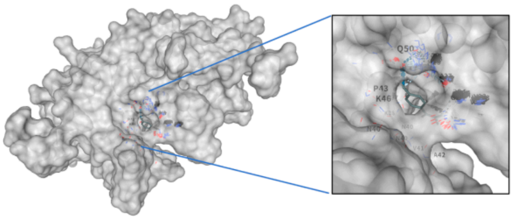

J

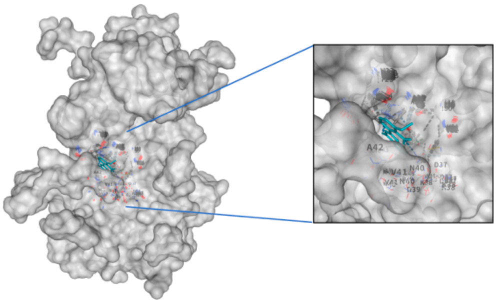

H

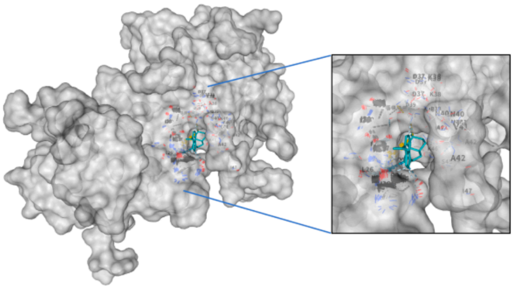

I

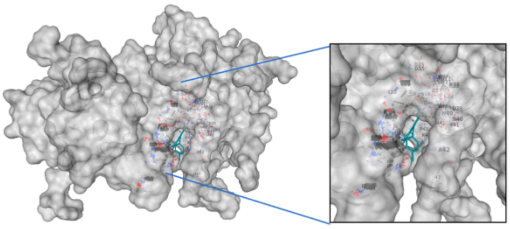

Supplement: Supplementary file 1 [file biomedicines-14-02096-s001.zip › Additional file 2.pdf]
